# 

**Published:** 1852-03

**Authors:** 


WHOLE SERIES—VOL. VIII, NO, VI.

VOL. IV.] MARCH, 1852. [No. 6

PUBLISHED EVERY OTHER MONTH.

### THE NORTH-WESTERN

### MEDICAL AND SURGICAL

### JOURNAL.

AT TWO DOLLARS PER ANUNM IN ADVANCE.

EDITED BY JOHN EVANS, M.D.,

PROFESSOR OF OBSTETRICS &C. IN RUSH MED. COL.; MEMBER OF THE AM. MED. ASSOCIATION; ONE OF THE PHYSICIANS TO THE ILL. GEN. HOSPITAL; FORMERLY SUP'T OF THE IND. HOSPITAL FOR THE INSANE, ETC., ETC.

CHICAGO AND INDIANAPOLIS:
PRINTED BY LANGDON & ROUNDS
161 Lake street, Chicago.
1852.

ENLARGED SERIES—VOL. VI, NO. VI.

